# "Is there nothing more practical than a good theory?": Why innovations and advances in health behavior change will arise if interventions are used to test and refine theory

**DOI:** 10.1186/1479-5868-1-11

**Published:** 2004-07-27

**Authors:** Alexander J Rothman

**Affiliations:** 1 Department of Psychology University of Minnesota Minneapolis, MN USA

## Abstract

Theoretical and practical innovations are needed if we are to advance efforts to persuade and enable people to make healthy changes in their behavior. In this paper, I propose that progress in our understanding of and ability to promote health behavior change depends upon greater interdependence in the research activities undertaken by basic and applied behavioral scientists. In particular, both theorists and interventionists need to treat a theory as a dynamic entity whose form and value rests upon it being rigorously applied, tested and refined in both the laboratory and the field. To this end, greater advantage needs to be taken of the opportunities that interventions afford for theory-testing and, moreover, the data generated by these activities need to stimulate and inform efforts to revise, refine, or reject theoretical principles.

## Background

Even with the dramatic advances in our understanding of the biological processes that determine health and illness, it has never been more clear that rates of disease morbidity and premature mortality reflect people's behavioral practices. [1] The benefits, both for individuals and the societies in which they live, that would come from systematic improvements in diet, physical activity, and use of substances such as tobacco, alcohol, and illicit drugs are tantalizing and provide ample motivation to develop initiatives to elicit changes in health behavior. Yet, health behavior change has proven a worthy adversary. Despite the commitment of considerable time and effort, innovations and advances in our ability to improve health behaviors have been modest. In particular, the specification of methods that produce sustained improvements in behavior have been elusive [2-5]. At the same time, innovations in theories of health behavior have also been modest. Investigators continue to advocate for a broad range of theories and there has been limited progress in demonstrating the unique value of any specific theory. [6-8]

Although there may be consensus in the professional community that there are considerable gaps in our understanding of health behavior change, critiques of the current state of affairs more often that not reflect the professional interests of the critic. Investigators who strive to specify the structural and psychological processes that regulate people's behavior lament the fact that too many interventions are not guided by a theoretical framework that specifies how they are supposed to elicit health behavior change. At the same time, investigators who design and implement health behavior interventions lament that the preponderance of theories of health behavior make it difficult to discern what factors are likely to be the most effective targets for intervention. Moreover, it is argued that theories are not sufficiently specified to determine when or how to modify factors that are to be targeted in an intervention.

Of course, concerns regarding the link between theory and practice are not new and efforts to address this problem have taken several forms. Considerable effort has been given to provide practitioners with a comprehensive and concise understanding of the array of theories that have been developed to address health behavior. [9] Moreover, conceptual frameworks such as PRECEDE-PROCEED [10] and Intervention Mapping [11] have been developed to provide investigators with a structured process to improve the accuracy and ease with which theoretical concepts are used to address a practical problem. In both cases, these efforts have targeted improving how theoretical principles are applied and, in doing so, have relied on the assumption that current theories of health behavior are useful and productive. Is this assumption valid? Could the often repeated plea for investigators to ground their intervention efforts in theory be a sign that there are significant limitations to the practical principles that can be derived from current theories of health behavior? If so, merely improving how people use theories will not be sufficient. What is needed is a shift in how we engage the interplay between theory and practice, with an emphasis placed on developing initiatives that target opportunities to develop, test, refine health behavior theory.

In this paper, I describe and advocate for a model of collaboration between basic and applied behavioral scientists. Although I recognize the value of improving the manner in which theoretical principles are matched to problems and methods, I propose that innovations in our understanding of and ability to promote health behavior change will not arise if theory is construed as a fixed entity that is delivered to interventionists for implementation. To date, although theories may fluctuate in their popularity, their properties have remained strikingly static over time. I believe greater attention must be paid to refining and, when necessary, rejecting theoretical principles. For this process to take shape, there needs to be an on-going series of exchanges between theorists and interventionists in which theory is treated as a dynamic entity whose value depends on it being not only applied and tested rigorously, but also refined based on the findings afforded by those tests.

A fundamental implication of this perspective is that improvements in both health behavior theory and intervention methods depend on each other. If investigators are more receptive to the opportunities interventions afford for theory testing, there will be a dramatic increase in data that can reveal the adequacies and inadequacies of a given theory. These data will, in turn, enable theorists to improve the quality of the theoretical models available to guide subsequent intervention efforts.

## Discussion

### When is an intervention effective?

Interventions are designed to address important practical problems (e.g., obesity) and thus their value is inextricably linked to their ability to alleviate the targeted problem. Interventions need to provide a meaningful return on the time, money, and effort invested such that the outcomes afforded by a intervention strategy are proportional to the resources utilized. Of course, determining what is a sufficient return on an investment can be a challenge. Small effects may be impressive if the intervention is directed at a construct or behavior that is considered difficult to move. [12] In addition, interventions can have minimal impact on an individual's behavior but when disseminated widely have a dramatic impact at the societal level. [13]

What conditions are likely to facilitate a successful intervention? Broadly speaking, an intervention is most likely to be effective if it is appropriately grounded in the practical problem targeted. [11] For example, consider an intervention to promote healthy food choices. The intervention design team must possess a clear understanding of who is engaging in the targeted behavior (e.g., who is making unhealthy food choices), the underlying nature of the behavior (e.g., the frequency and function of food choices), and the context in which the behavior is performed (e.g., where and with whom do people make choices about food). In a similar manner, the intervention needs to be appropriately grounded in the biological, structural and psychological processes that shape and regulate people's behavioral practices. [14-16] For example, the expected value of altering a feature of the environment in which people make food choices (e.g., increasing the cost of high-fat foods) is predicated on the assumption that the intervention will directly, or indirectly through an intervening construct, influence people's food choice in that setting.

Health behavior theories provide an explicit statement of the structural and psychological processes that are hypothesized to regulate behavior (e.g., increasing the cost of high-fat foods will curtail consumption of these foods by making it more aversive or, perhaps, more difficult to purchase them). If theories describe the factors that guide people's behavior and justify how an intervention is designed and implemented, interventionists depend on the quality and predictive value of a theory. What determines a theory's value? From the perspective of a theoretician, a theory's value rests on its ability to provide an accurate account of the factors that regulate people's behavior. [17] Although investigators may recognize that behavior is affected by factors at different levels of analysis (i.e., biological, psychological, social, environmental), a theory's value is not necessarily predicated on its ability to provide linkages across these levels. Because of this emphasis, theory testing tends to occur in controlled contexts, typically a laboratory setting, that afford the social and behavioral version of a Petrie dish. This approach allows investigators to observe the relation between a given set of constructs with greater precision, but it renders the generalizability and strength of the observed effect difficult to discern. For example, investigators may determine that focusing people's attention on the undesirable aspects of an object increases their interest in avoiding it, but be unable to specify the conditions under which this relation is and is not most likely to obtain.

From the perspective of an interventionist, the accuracy of the relations specified in a theory is an important but not sufficient determinant of its value. Interventionists need theories that are accurate *and *applicable; that specify not only the relation between two constructs, but also whether that relation does or does not change across contexts (e.g., does the impact of risk perceptions on behavior differ whether one is examining decisions to test for radon or to start smoking?). Given a set of a factors hypothesized to regulate people's behavior, interventionists need to be able to discern which of these factors are the most appropriate targets for intervention. In fact, a common complaint regarding theories is that they are not useful (See Jeffery, this issue). A theory may specify a host of factors that regulate a person's behavior, but in the absence of information regarding the relative importance of each factor leave an interventionist unsure as to where to direct her or his resources. For example, the Theory of Planned Behavior [18] and Theory of Reasoned Action [19] propose that people's attitudes toward the behavior and their perceived subjective norm regarding the behavior are critical determinants of behavior (albeit mediated by behavioral intention), but the relative contribution of these constructs is allowed to fluctuate from setting to setting. In any given context, it is unclear how to determine *a priori *which set of constructs should be prioritized as a target for intervention. The interest interventionists have shown in stage-based models of health behavior may reflect the fact that the models attempt to specify the conditions under which specific constructs affect behavioral decisions. [8]

Little guidance is also given as to how or even whether critical constructs can be manipulated. For example, my colleagues and I have proposed that satisfaction with the outcomes afforded by a pattern of behavior is a critical determinant of behavioral maintenance. [20,21] Claims such as this are typically predicated on evidence that measures of a construct, in this case satisfaction, uniquely predict a behavioral outcome. Yet, the observation that someone who is satisfied is more likely to sustain a pattern of behavior does not indicate what causes someone to be satisfied and, thus, little guidance is given as to what can be done to heighten the satisfaction people derive from changes in their behavior. In the absence of this type of information, interventionists may find little difference between developing intervention strategies that are or are not grounded in a health behavior theory. In fact, given these practical needs, it is not be surprising that interventionists are more likely to rely on health behavior theories (e.g., Social Cognitive Theory [22]) that specify the determinants of its primary constructs and thus provide guidance as to how to construct an intervention protocol.

### Breakdown in the evolution of health behavior theories

If the design and implementation of intervention strategies rely on assumptions regarding the factors that regulate people's behavior, why haven't current theories of health behavior evolved in ways that would enable them to more effectively guide intervention development? I believe the critical problem is that there has been a breakdown in the relation between basic and applied scientists who study health behavior. [23] As scholars such as Kurt Lewin [24] have asserted, the development and specification of theories of human behavior depend upon an iterative series of research activities in which theoretical principles initially formulated by basic behavioral scientists are tested and evaluated by applied behavior scientists. These tests provide critical information that enables basic scientists to revise, refine, or reject their initial principles. Moreover, an applied setting can afford investigators the opportunity to assess the relative impact of different processes hypothesized to regulate people's behavior. It is through this on-going cycle of specification, application, and evaluation that accurate and applicable theoretical models arise.

To the extent that behavioral theories are not tested in complex social settings such as those afforded by interventions to change health practices, the process by which theories develop is curtailed. Because the manner in which a theory is specified reflects, in part, the contexts in which it has been operationalized and tested, theories that are tested primarily in tightly controlled laboratory settings will likely be characterized by a rich description of the myriad of factors that could affect people's behavioral choices. The laboratory setting allows investigators to minimize noise and potential confounding or moderating factors and thus optimizes their ability to detect processes that *can *affect people's behavior without determining whether, in a more complex setting, they *do *affect behavior. [17] Thus, in the absence of initiatives that empirically test theoretical principles in complex social environments, investigators run the risk of developing a "hot house" theory of health behavior that has limited practical value.

Interventions afford an invaluable opportunity to discern the context dependence of causal relations that have been revealed in the laboratory. Some factors may be shown to always be critical, whereas others may be critical only under certain conditions. [25] For example, self-efficacy may be a critical determinant of the decision to initiate a new pattern of behavior, but have a limited impact on the decision to maintain that behavior over time. [21] It is critical to understand that restricting the conditions under which a construct affects behavior does not mean that a given factor is not important. Information that would help delimit these conditions would enable theorists to develop more precise models.

### The case for why interventions should be more receptive to theory

There are two sets of reasons why we must take better advantage of the opportunities interventions provide to implement and test theories of health behavior. One set focuses on what theory can do to improve the implementation and evaluation of an intervention, whereas the other set focuses on how interventions can be used to improve the accuracy and quality of prevailing health behavior theories. First, by grounding their work on theoretical principles regarding processes that regulate people's behavior, investigators can readily specify the critical assumptions that underlie their intervention protocol. These formal statements of cause and effect relations not only provide a clear justification for the proposed research activities (i.e., why an investigator believes a given intervention strategy will be effective), but also increase the likelihood that the proposed methodology will allow the investigator to detect whether and why the intervention had its intended effect. [10,11]

When faced with unambiguous evidence of a successful intervention effect, investigators might be able to move forward without knowing why the intervention was effective. However, more often than not, investigators are faced with the task of determining why an intervention failed to produced the desired effect or why it worked under a limited set of conditions. An *a priori *set of theoretical principles can provide an important conceptual and analytic framework for determining why an intervention was ineffective. In particular, it increases the likelihood that investigators have not only identified the constructs that may determine whether an intervention will prove effective, but also assessed them at the appropriate points in the decision process.

The second set of reasons why interventions should take advantage of opportunities to test theories of health behavior is that by providing a context in which some or all of the facets of a theory can be tested, interventionists are in a position to generate evidence that will enhance the accuracy and applicability of theory and thus, over time, improve the quality of the theories to which interventionists can turn. By systematically testing principles specified in health behavior theories, investigators are able to not only verify the accuracy of these predictions, but also develop a better understanding of their practical value. Across studies, evidence should accumulate that will allow investigators to differentiate between factors that should and should not be targeted for intervention. Because current theories of health behavior often provide a list of factors that may affect behavior, the set of potential mediating variables suggested by a theory may pose a daunting if not untenable measurement burden. However, the implementation of consistent and methodologically sound assessment of these factors should provide the empirical evidence needed to constrain and prioritize the variables on that list.

The characteristics of intervention strategies that prove to be effective should also provide investigators with a better understanding of the determinants of a given construct. As was previously mentioned, theories may propose that a construct (e.g., satisfaction) is a critical determinant of decisions to maintain a new pattern of behavior, but provide limited guidance as to how to alter people's standing on that construct (e.g., how to help feel satisfied with the outcomes afforded by their new behavior). [21] An intervention protocol that is shown to successfully heighten people's satisfaction with process and outcomes associated with weight loss not only has clear practical value, but also can shed light on the process by which people determine whether they are satisfied with their experiences. If theorists can develop a more detailed account of the processes that shape the primary constructs identified in a health behavior theory, interventionists will find that theories can provide a more useful set of guidelines for how to develop strategies to target these constructs.

Testing theoretical principles across a diverse array of settings and populations will also enable investigators to better specify the scope of a theory. Although interventions provide a wonderful opportunity to test theoretical principles in diverse samples and settings, formal and appropriately powered tests of moderators can put a considerable strain on sample size and resources. However, if investigators have appropriately assessed the critical constructs, systematic comparisons can be drawn across studies that taken together have tested a theoretical principle across a range of settings or people. The increase in public access to data sets should facilitate opportunities for this type of comparisons. With the information that is gleaned from these types of activities, it should be easier to determine which moderators are worth testing in a single, appropriately powered study design.

The identification of situational or personal factors that moderate the impact of a theoretical principal can be indicative of a number of different scenarios. For example, what might one conclude if an intervention that promoted the health benefits of eating a balanced diet altered the eating habits of college students but not those of high school students? It could indicate that health benefits do not affect what high school students choose to eat. Alternatively, it might be that high school students *are *responsive to perceptions of the health benefits afforded by a balanced diet, but that other factors (e.g., control over access to food) preclude them from acting on those beliefs. The practical and theoretical conclusions that can be drawn from the identification of moderating factors are dramatically increased if investigators can identify the causal processes that underlie the observed impact of the moderator. In particular, can investigators discern whether the moderated effect was obtained because the moderator altered the ability of the intervention strategy to change the proposed mediating construct (e.g., the intervention raised perceptions of the health benefits held by college but not high school students) or because it altered the effect the mediator has on the primary outcome measure (e.g., perceived health benefits predicted the eating habits of college but not high school students)? Greater attention to the causal processes invoked by a moderator may also help investigators grapple with the daunting number of potential moderators. It is quite possible that moderators that differ at the level of description (e.g., gender, ethnicity) can be accounted for by the same underlying process.

Finally, it is important to recognize that progress in theory development can arise from the failure to obtain evidence in support of a specific prediction. Empirical evidence that provides investigators with a better sense of the potential factors that do not affect health practices will allow them to reduce the number of constructs (and, in time, theories) invoked to predict and explain health behavior.

### What can be done to make interventions more theory-friendly?

If one assumes that there is interest in rendering interventions more receptive to theory-testing, what can be done to enhance an intervention's ability to assess principles derived from current health behavior theories? One issue is the appropriate evaluation of the critical manipulation(s) imbedded in the intervention. Any conclusions that can be drawn from the intervention, regardless of whether it reveals the predicted pattern of results, is predicated on the success with which the independent variable was manipulated. To this end, investigators need to at least consider assessing several constructs: the degree to which the intervention was implemented (e.g., did the interventionists consistently provide participants with the intervention exercises?), the degree to which participants correctly identified the emphasis of the intervention (e.g., did participants assigned to the optimistic outcome condition report their was a greater emphasis on favorable outcomes than did those assigned to the control condition?), and finally the degree to which the intervention altered the targeted set of opportunities, thoughts or feelings (e.g., did those assigned to the optimistic outcome condition develop more favorable expectations regarding the benefits afforded by behavior change than did those assigned to the control condition?).

Although it is important that interventionists explicitly specify the constructs that determine the influence of the intervention on participant behavior, the quality of the evidence that can be gathered depends on the assessment procedures that are utilized. The persuasiveness of any claims regarding the importance (or lack of importance) of a particular construct is contingent on the use of measures that have been shown to be reliable and valid. Given that many of the constructs specified in theories of health behavior are conceptually similar, it is difficult to draw strong conclusions regarding the specific contributions of different variables in the absence of well-designed measures. [26,27] In addition, the inclusion of a pool of potential mediators enables the investigators to make stronger claims as he or she can demonstrate that not only does the construct specified in the model serve as a mediator but that other factors do not operate as mediators.

Adequately testing basic principles also depends on a well-timed assessment schedule. Assessments are often too infrequent to detect meaningful changes on the construct. This is particularly true if the constructs of interest are psychological states that both affect and are affected by behavioral practices. However, specifying the optimal time to assess the primary constructs can be difficult. To the extent that one wants to determine whether an intervention strategy (e.g., a tailored message about dietary changes) alters the predicted mediating variable (e.g., willingness to modify one's diet), one might consider minimizing the length of time between the delivery of the intervention and the assessment of the mediator. However, at the same time, interest in the association between the hypothesized mediator and the outcome variable (e.g., change in diet) would also benefit from a shorter window of time between the two assessments. In many cases, the length of these two time windows are inversely related to each other and thus efforts to improve the chance of detecting one relation may hinder effects to detect the other. Of course, there are practical constraints on an investigator's ability to adequately assess constructs. What is needed is for investigators to take advantage of the measurement and testing opportunities when they do arise. Although what can be concluded from any single assessment effort may be limited, the cumulative impact of well designed tests of a theoretical principle can be substantial. If investigators consistently wait for another time or another investigator to conduct the relevant assessments, innovations in theory and practice will continue to be slow.

As interventionists specify the degree to which a given study can test all or a facet of a given theory, they are more likely to articulate the contribution a proposed study could make to the empirical literature. This process not only makes the justification for the intervention clear, but also improves the likelihood that investigators will recognize when their and their colleagues' efforts have focused consistently on a single or limited aspect of a given theory. Research activities motivated by the Transtheoretical Model [28] provide an excellent example of a domain where researchers have consistently relied on a limited number of methodological strategies and thus, despite an enormous amount of research activity, provided a very narrow test of the theory. [8]

The commitment of time and effort to using interventions to test theoretical principles will in the end be for naught, if there is not an equal commitment to the dissemination of the findings generated by these activities. In particular, investigators who are engaged in the development of health behavior theories must take advantage of the information afforded by intervention activities and demonstrate that they are responsive to this information as they refine and revise their theories. Enhanced communication should also provide an opportunity for basic and applied behavioral scientists to recognize the strengths and weakness of current theories of health behavior and thus help formulate a fuller understanding of what needs to be done to improve the quality of our theories.

## Summary

### With an eye toward the future

Although Lewin may have been right that there is "nothing more practical than a good theory" (p.169; [24]), his dictum rests on the assumption that good theories are available to address practical problems. The development of "good" theories – that is, theories that are both accurate and applicable – has been hindered by a breakdown in the on-going collaboration between basic and applied behavioral scientists. Research and professional activities that are able to foster a stronger sense of interdependence between these two groups are likely to provide a base for collaboration and, in turn, a opportunity for innovation. If critical advances in health behavior theory depend on an iterative process by which theoreticians and interventionists cooperate in the testing and evaluation of theoretical principles, individuals in both camps need to not only recognize the goals and values of each group, but also trust each other's ability to advance our understanding of both theory and practice.

## Competing Interests

None declared.
